# Cytogenetics and Molecular Genetics of Myxoid Soft-Tissue Sarcomas

**DOI:** 10.4061/2011/497148

**Published:** 2011-07-28

**Authors:** Jun Nishio, Hiroshi Iwasaki, Kazuki Nabeshima, Masatoshi Naito

**Affiliations:** ^1^Department of Orthopaedic Surgery, Faculty of Medicine, Fukuoka University, 7-45-1 Nanakuma, Jonan-ku, Fukuoka 814-0180, Japan; ^2^Department of Pathology, Faculty of Medicine, Fukuoka University, Fukuoka 814-0180, Japan

## Abstract

Myxoid soft-tissue sarcomas represent a heterogeneous group of mesenchymal tumors characterized by a predominantly myxoid matrix, including myxoid liposarcoma (MLS), low-grade fibromyxoid sarcoma (LGFMS), extraskeletal myxoid chondrosarcoma (EMC), myxofibrosarcoma, myxoinflammatory fibroblastic sarcoma (MIFS), and myxoid dermatofibrosarcoma protuberans (DFSP). Cytogenetic and molecular genetic analyses have shown that many of these sarcomas are characterized by recurrent chromosomal translocations resulting in highly specific fusion genes (e.g., *FUS-DDIT3* in MLS, *FUS-CREB3L2* in LGFMS, *EWSR1-NR4A3* in EMC, and *COL1A1-PDGFB* in myxoid DFSP). Moreover, recent molecular analysis has demonstrated a translocation *t*(1; 10)(p22; q24) resulting in transcriptional upregulation of *FGF8* and *NPM3* in MIFS. Most recently, the presence of *TGFBR3* and *MGEA5* rearrangements has been identified in a subset of MIFS. These genetic alterations can be utilized as an adjunct in diagnostically challenging cases. In contrast, most myxofibrosarcomas have complex karyotypes lacking specific genetic alterations. This paper focuses on the cytogenetic and molecular genetic findings of myxoid soft-tissue sarcomas as well as their clinicopathological characteristics.

## 1. Introduction

Myxoid soft-tissue sarcomas encompass a heterogeneous group of rare tumors characterized by a marked abundance of mucoid/myxoid extracellular matrix. The main clinicopathological entities in this group are myxoid liposarcoma, low-grade fibromyxoid sarcoma, extraskeletal myxoid chondrosarcoma, myxofibrosarcoma, myxoinflammatory fibroblastic sarcoma, and myxoid dermatofibrosarcoma protuberans [1–4]. The correct classification of these sarcomas is important because of their distinct biological behaviors and potentially different treatments. However, it is often difficult to set apart many of these sarcomas due to overlapping histological features and lack of a distinct immunohistochemical profile. Moreover, the use of core needle biopsies to diagnose these sarcomas has become increasingly common, and this shift has created additional challenges.

 Cytogenetic and molecular genetic assays are routinely used for diagnostic and prognostic purposes in molecular pathology laboratories [5]. Many of myxoid soft-tissue sarcomas are characterized by recurrent chromosomal translocations resulting in highly specific fusion genes [6, 7]. Advances in knowledge of the genetics of these sarcomas are leading to more accurate diagnosis. This paper reviews the cytogenetic and molecular genetic findings in these sarcoma types and their relationship with clinicopathological features. The consistent genetic alterations are summarized in Table 1.

## 2. Approaches to the Genetics of Soft-Tissue Sarcomas

Conventional karyotyping is the most comprehensive method for spotting the various translocations and other structural or numerical aberrations. It is dependent on the availability of fresh, sterile tumor tissue, the success of tumor cell growth in culture, and quality of metaphase cell preparations. When dividing cells are not available for cytogenetic studies, molecular approaches such as fluorescence in situ hybridization (FISH), comparative genomic hybridization (CGH), reverse transcriptase-polymerase chain reaction (RT-PCR), or gene expression microarray can be used to evaluate genetic alterations.

 FISH is the most helpful method for identifying specific gene rearrangements. It is more adaptable to formalin-fixed, paraffin-embedded tissues although imprint slides are preferred. Interphase FISH is particularly useful to assess intratumoral genetic heterogeneity as long as adequate combination of probes are used. FISH probes are readily available for a variety of relevant gene targets, including *DDIT3* (12q13), *FUS* (16p11), *EWSR1* (22q12), *FKHR *(13q14), *SYT* (18q11.2), and *ALK* (2p23) (Abbott Molecular Inc., Des Plaines, Ill, USA). It has been realized that FISH is a valuable adjunct in the diagnosis of myxoid soft-tissue tumors [8].

 CGH is a method for genome-wide analysis of DNA sequence copy number in a single experiment. It maps the origins of amplified and deleted DNA sequences on normal chromosomes, thereby highlighting regions harboring potential oncogenes and tumor suppressor genes. However, CGH cannot detect rearrangements such as balanced translocations or inversions. Recently, a higher resolution version of CGH, so-called array CGH, has been made available. A distinct advantage of array CGH is the ability to directly map the copy number changes to the genome sequence. Similar to array CGH, single nucleotide polymorphism (SNP) array is capable of identifying small regions of chromosomal gains and losses at a high resolution. Also, SNP array can provide information regarding loss of heterozygosity.

 RT-PCR is the most sensitive method to detect small numbers of translocation-bearing cells that are mixed within a tissue consisting of largely nonneoplastic cells. Sensitivity levels of 1 in a 100,000 cells are typically achieved. It may be suitable for the detection or monitoring of minimal residual disease [9]. However, the diagnostic success rate is variable and dependent on multiple factors. First, RNA quality may be inadequate because of RNA degradation. The second impediment of this methodology is the high risk of reagent contamination, mainly with PCR products, particularly in small laboratory spaces.

 Microarray is a method for genome-wide monitoring of gene expression in a single experiment. A variety of commercial and noncommercial platforms can be used to perform global gene expression profiling. It is hoped that application of this technology will afford increased understanding of sarcoma biology and facilitate the development of new diagnostic markers and therapeutic agents [10–12].

 Approximately one-third of all soft issue sarcomas exhibit a nonrandom chromosomal translocation. In addition, a subset of soft-tissue tumors carries specific oncogenic mutations (e.g., *KIT* or *PDGFRA* mutations in gastrointestinal stromal tumor). FISH and RT-PCR are commonly applied for the detection of specific genetic alterations in the differential diagnosis of soft-tissue sarcomas.

## 3. Myxoid Liposarcoma

The working group of the World Health Organization (WHO) for classification of tumors of soft-tissue and bone combined myxoid and round cell liposarcomas under myxoid liposarcoma (MLS) [13]. MLS is the second most common subtype of liposarcoma, representing approximately one-third of all liposarcomas. MLS occurs predominantly in the deep soft-tissues of lower extremities and has a peak incidence in the fourth and fifth decades of life with no gender predilection. Pure MLS is considered low-grade and has a 5-year survival rate of 90% [14]. In contrast, MLS containing a greater than 5% round cell component is considered high-grade and has a worse prognosis. The clinical outcome of multifocal MLS is poor [15]. In contrast to other soft-tissue sarcomas, MLS tends to metastasize to unusual sites such as retroperitoneum, opposite extremity, and bone.

**Figure 1:**
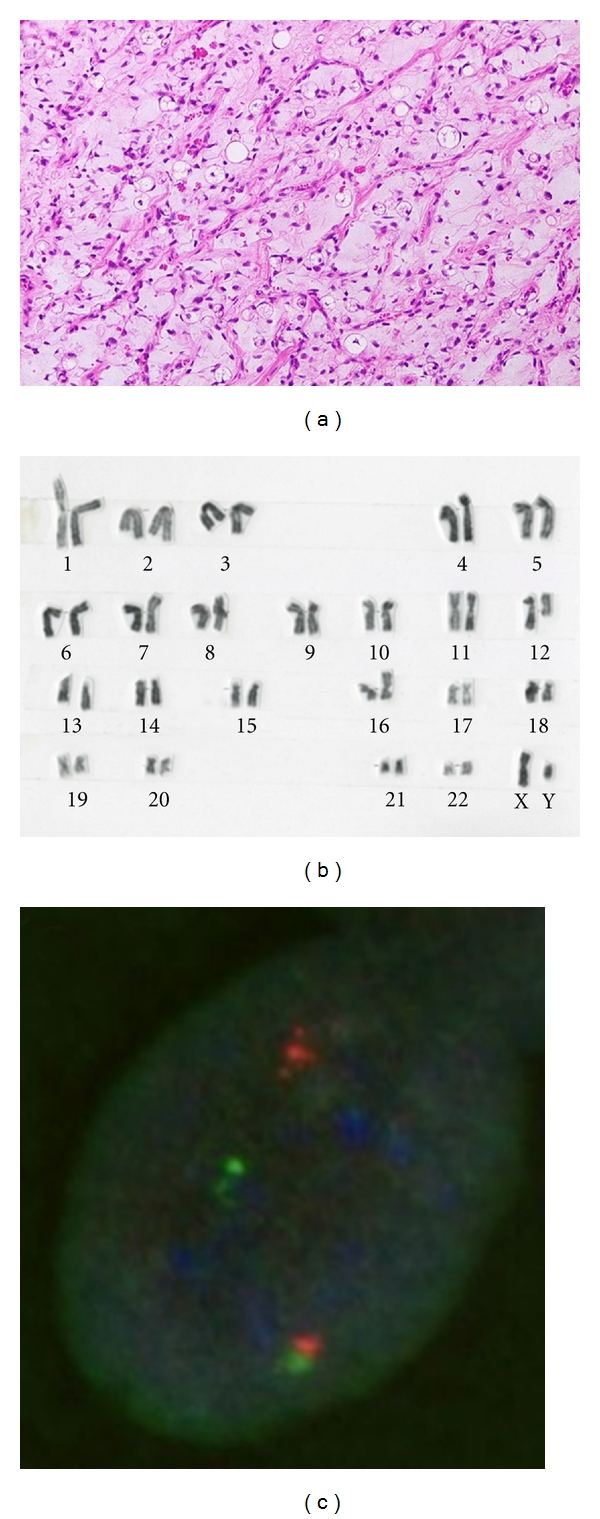
(a) Myxoid liposarcoma with a myxoid background containing a delicate arborizing capillary vascular network, small uniform mesenchymal cells, and lipoblasts. (b) G-banded karyotype showing a 12; 16 translocation as the sole aberration. (c) Fluorescence in situ hybridization analysis using a *DDIT3* (12q13) break-apart probe shows a split of the orange and green signals, indicating a disruption of the *DDIT3* locus.

Histologically, pure MLS is composed of primitive mesenchymal cells in a myxoid matrix, often featuring mucinous pools (Figure 1(a)). Lipoblasts are most often univacuolated, small, and tend to cluster around vessels or at the periphery of the lesion. A delicate plexiform capillary vascular network is present and provides an important clue for distinguishing MLS from intramuscular myxoma [16]. A subset of MLS shows histological progression to hypercellular or round cell morphology. The round cell areas are characterized by solid sheets of primitive round cells with a high nuclear/cytoplasmic ratio and a prominent nucleolus. Pure round cell liposarcoma is extremely rare and may be confused with other round cell sarcomas such as Ewing sarcoma/primitive neuroectodermal tumor, rhabdomyosarcoma, and poorly differentiated synovial sarcoma.

MLS is characterized by a recurrent translocation *t*(12; 16)(q13; p11) in more than 90% of cases (Figure 1(b)), which fuses the 5′ portion of the *FUS* gene on chromosome 16 with entire reading frame of the *DDIT3* gene on chromosome 12 [17–19]. A small percentage of cases carry a variant translocation *t*(12; 22)(q13; q12) resulting in an *EWSR1-DDIT3* fusion gene [15, 20–28]. The presence of these translocations and molecular alterations is highly sensitive and specific for MLS. Therefore, cytogenetics is an excellent analytic method for the initial workup of a suspected MLS. FISH and RT-PCR can also be used to provide support for the diagnosis of MLS (Figure 1(c)) [8, 29–32]. In addition, several nonrandom secondary alterations have been identified, including 6q deletion, isochromosome 7q10, trisomy 8, and unbalanced 1; 16 translocation [17, 24, 33–35]. Conventional and array CGH studies have shown gains of 8p21–23, 8q, and 13q [36–38].

To date, 12 *FUS-DDIT3* and four *EWSR1-DDIT3* variants of fusion transcripts have been described in MLS [22, 26, 28, 39, 40]. Most cases of MLS are one of three different *FUS-DDIT3* fusion transcript types, including varying portions of *FUS*. The *FUS-DDIT3* fusion transcript type does not appear to have a significant impact on clinical outcome [22, 26]. On the other hand, Suzuki et al. [28] reported that MLS with a type 1 *EWSR1-DDIT3* fusion transcript may show more favorable clinical behavior than MLS with other types of fusion transcripts. Interestingly, clinical data suggest that the fusion transcript type may influence response to therapy with trabectedin [41].

 Several receptor tyrosine kinases (RTKs) are highly expressed in MLS, including RET, MET, and IGF1R [42, 43]. These RTKs promote cell survival and cell proliferation through the PI3K/AKT and the Ras-Raf-ERK/MAPK pathways [42]. Recently, Barretina et al. [44] demonstrated that mutation of *PIK3CA*, encoding the catalytic subunit of PI3K, is associated with AKT activation and poor clinical outcome. AKT activation functions as a master switch to generate a plethora of intracellular signals and intracellular responses and is more frequent in the round cell variant [43]. It has also been shown that the NF-*κ*B pathway is highly active in MLS [40]. Moreover, Göransson et al. [45] showed that NF-*κ*B is a major factor controlling *IL8* transcription in FUS-DDIT3-expressing cells. NF-*κ*B is an inducible cellular transcription factor that regulates a variety of cellular genes, including those involved in immune regulation, inflammation, cell survival, and cell proliferation. These findings will help to develop new potential therapeutic strategies for MLS patients with advanced disease.

## 4. Low-Grade Fibromyxoid Sarcoma

Low-grade fibromyxoid sarcoma (LGFMS), first described by Evans [46] in 1987, is a rare but distinctive fibromyxoid variant of fibrosarcoma. It includes the tumor originally designed as hyalinizing spindle cell tumor with giant rosettes [47]. LGFMS occurs primarily in young to middle-aged adults with a male predominance, but this tumor may affect children [48, 49]. LGFMS typically presents as a slowly growing, painless mass in the deep soft-tissues of lower extremities or trunk. Local recurrence and metastatic rates are 9%–21% and 6%–27%, respectively [49, 50]. The overall prognosis for superficial LGFMS appears to be better than that for deep LGFMS [48].

**Figure 2:**
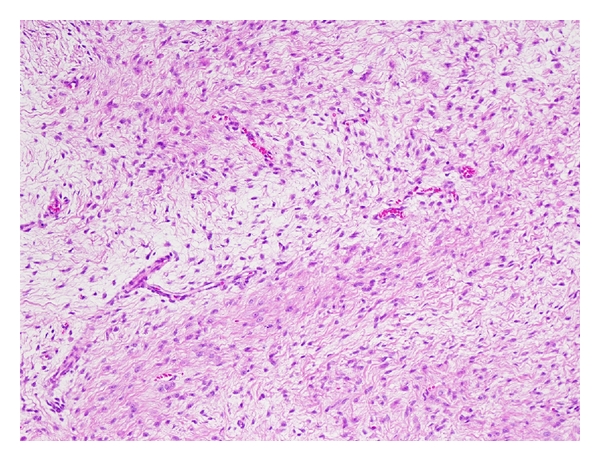
Low-grade fibromyxoid sarcoma with alternating fibrous and myxoid areas.

 Histologically, LGFMS shows alternating fibrous and myxoid areas with bland spindle-shaped cells arranged in a whorled pattern (Figure 2). Cellularity is variable but generally low and mitoses are scarce. There is often a prominent network of branching capillary-sized blood vessels reminiscent of myxoid liposarcoma. Approximately 40% of cases have giant collagen rosettes characterized by a central zone of eosinophilic collagen surrounded by a palisade of round to oval tumor cells [13]. This variant was originally termed hyalinizing spindle cell tumor with giant rosettes [47]. Immunohistochemically, the tumor cells are diffusely positive for vimentin and focally for epithelial membrane antigen (EMA) [48, 50]. Immunostains for S-100 protein, desmin, and CD34 are typically negative.

 LGFMS is characterized by a recurrent balanced translocation *t*(7; 16)(q34; p11) resulting in an *FUS-CREB3L2* fusion gene [50–53]. This same translocation was identified in cases of hyalinizing spindle cell tumor with giant rosettes [54, 55], suggesting a pathogenetic link between these two entities. A small percentage of cases carry a variant translocation *t*(11; 16)(p11; p11) leading to a fusion of the *FUS* and *CREB3L1* genes [50, 53]. Interestingly, supernumerary ring chromosomes have been observed as the sole anomaly in a subset of LGFMS [52, 56, 57]. FISH and CGH studies have demonstrated that ring chromosomes are composed of material from chromosomes 7 and 16 [56, 57]. Bartuma et al. [57] showed that the *FUS-CREB3L2* fusion gene can be present in ring chromosomes.

 The breakpoints in the fusion transcripts are mostly at exon 6 or 7 of *FUS* and exon 5 of *CREB3L2* or *CREB3L1* [50–53, 58]. CREB3L2 is a member of CREB3 family of transcription factors and contains a basic DNA-binding and leucine zipper dimerization domain, highly similar to that in CREB3L1. Panagopoulos et al. [59] suggested that the FUS-CREB3L2 fusion protein is a more potent transcriptional activator than the native CREB3L2 and may contribute to the pathogenesis of LGFMS through the deregulation of its target genes. The molecular variability of fusion transcripts in LGFMS does not appear to have a significant impact on microscopic appearances or clinical outcome [53].

## 5. Extraskeletal Myxoid Chondrosarcoma

Extraskeletal myxoid chondrosarcoma (EMC) is categorized by the WHO as a tumor of uncertain differentiation, because there is a paucity of convincing evidence of cartilaginous differentiation [13]. Most EMCs arise in the deep soft-tissues of the proximal extremities and limb girdles, especially the thigh and popliteal fossa, similar to MLS. EMC has a peak incidence in the fifth and six decades of life with a male predominance. Only a few cases have been encountered in children and adolescents [60–62]. Patients typically present with a slowly growing mass that causes pain or tenderness in approximately one-third of cases [16]. Local recurrence and metastatic rates are 48% and 46%, respectively [61]. EMC has a 10-year survival rate of 63%–88%, but a 10-year disease-free survival is much lower, ranging from 14% to 36% [61, 63–66]. Large tumor size (especially >10 cm), advanced age, and proximal tumor location appear to be poor prognostic factors in EMC [61, 63, 67].

**Figure 3:**
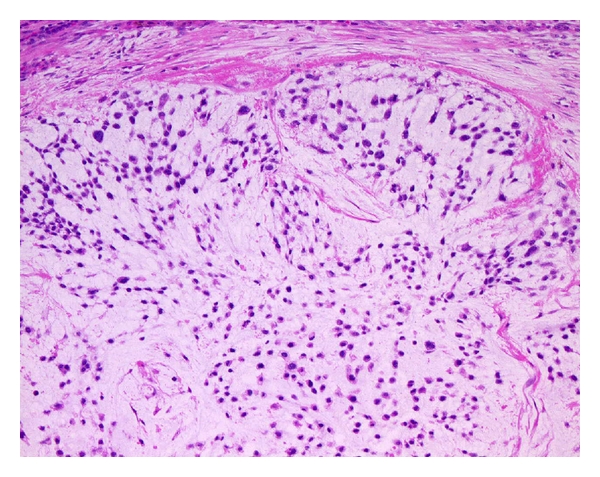
Extraskeletal myxoid chondrosarcoma with an abundant myxoid matrix containing round or slightly elongated cells with small hyperchromatic nuclei.

 Histologically, EMC is characterized by multinodular growth of a cord-like or lace-like arrangement of round or slightly elongated cells in an abundant myxoid matrix (Figure 3). The tumor cells have small hyperchromatic nuclei and a narrow rim of deeply eosinophilic cytoplasm. Occasional cells show cytoplasmic vacuolization [16]. Mitotic figures are rare in most cases. In contrast to the bland-looking or low-grade morphology, cellular or high-grade EMC has also been described [61, 68, 69]. Some authors have suggested that the cellular or high-grade EMC is likely to have a worse prognosis than conventional EMC [63, 68, 70] although its prognostic significance has not yet been established [67]. Immunohistochemically, vimentin is the only marker consistently positive in EMC. S-100 protein is expressed in approximately 30% of cases [67], often with focal and weak immunoreactivity. Only a small percentage of cases may show scattered cells that are EMA positive [67]. Recent immunohistochemical and ultrastructural studies have demonstrated that some EMCs may have neuroendocrine differentiation [63, 69, 71].

 EMC is characterized by a recurrent translocation *t*(9; 22)(q22; q12) in approximately 75% of cases, which fuses the *EWSR1* gene on 22q12 with the *NR4A3* gene on 9q22 [72–78]. A second variant translocation, *t*(9; 17)(q22; q11), has been detected in approximately 15% of EMC and results in a *TAF15-NR4A3* fusion gene [78–82]. In addition, two additional variant translocations, *t*(9; 15)(q22; q21) resulting in a *TCF12-NR4A3* fusion gene and *t*(3; 9)(q12; q22) resulting in a *TFG-NR4A3* fusion gene, have also been identified, each only in a single case [83, 84]. Because these fusion genes have not yet been described in any other tumor type, they represent useful diagnostic markers. Moreover, several nonrandom secondary alterations have been identified in approximately 50% of cytogenetically analyzed cases, including gain of 1q and trisomy for chromosomes 7, 8, 12, and 19 [77, 78]. The biological significance of these chromosomal alterations remains unknown.

 Two main *EWSR1-NR4A3* fusion transcript types have been reported for the *t*(9; 22)(q22; q12) in EMC [69, 77, 78]. The most common fusion transcript contains exon 12 of *EWSR1* fused to exon 3 of *NR4A3* (type 1), whereas exon 7 of *EWSR1* is fused to exon 2 of *NR4A3* in the type 2 fusion transcript. In the *TAF15-NR4A3* fusion transcript, exon 6 of *TAF15* is fused exclusively to exon 3 of *NR4A3* [77]. NR4A3 is a member of NR4A subfamily within the nuclear receptor superfamily and contains a zinc finger DNA-binding domain. The EWSR1-NR4A3 fusion protein is thought to function as a potent transcriptional activator for *NR4A3*-target genes [85, 86]. It has also been shown that the TAF15-NR4A3 fusion protein functions a strong transcriptional activator [87]. It is unclear whether the fusion transcript type is associated with particular morphological features or clinical outcome.

 Gene expression profiling studies of EMC have revealed overexpression of the *CHI3L1*, *METTL1*, *RELB*, *MYB*,* NMB*, *DKK1*, *DNER*, *CLCN3*, *DEF6*, *NDRG2*, and *PPARG* genes [78, 88, 89]. In addition, several genes encoding neural-neuroendocrine markers have been expressed, including *SCG2*, *NEF3*, *GFAP*, *GAD2*, *ENO2*, *SYP*, *CHGA*, *NEF3*, and *INSM1* [78, 88]. *CHI3L1* encodes a glycoprotein member of the glycosyl hydrolase 18 family, which is secreted by activated chondrocytes, macrophages, neutrophils, and synovial cells. Sjögren et al. [78] suggested that CHI3L1 may be useful as a serum marker monitoring disease progression in EMC patients. NMB is a member of bombesin-related peptide family in mammals and a secreted protein involved in stimulation of smooth muscle contraction [90]. Subramanian et al. [88] suggested that NMB may prove to be a serological marker of EMC recurrence. *DKK1* encodes a protein that is a member of the dickkopf family. DKK1 is involved in embryonic development through its inhibition of the WNT signaling pathway. Because DKK1 is a secreted protein, it may serve as a prognostic marker for evaluation of EMC. *PPARG* encodes a member of the peroxisome proliferator-activated receptor subfamily of nuclear receptors. PPARG is known as a regulator of adipocytic differentiation [91]. Interestingly, Filion et al. [89] demonstrated that *PPARG* is the first direct transcriptional target of the EWSR1-NR4A3 fusion protein. These findings will lead to the development of molecularly targeted therapies for patients with advanced EMC.

## 6. Myxofibrosarcoma

Myxofibrosarcoma, formerly known as myxoid malignant fibrous histiocytoma (MFH), is now defined as a distinct histological entity [13]. It is one of the most common soft-tissue sarcomas in elderly patients. Most myxofibrosarcomas arise in the dermal and subcutaneous tissues of the limbs (especially lower limbs) and limb girdles. Myxofibrosarcoma has a peak incidence in the sixth to eighth decades of life with a slight male predominance. Patients typically present with a slowly growing, painless mass. Recently, an epithelioid variant of myxofibrosarcoma with an aggressive course has been described [92].

Grading of myxofibrosarcoma is somewhat controversial. Myxofibrosarcoma has been subdivided into three or four grades based on the degree of cellularity, nuclear pleomorphism, and mitotic activity [93, 94]. Local recurrences occur in up to 50% to 60% of cases [93–95], irrespective of histological grade. Whereas low-grade myxofibrosarcomas usually do not metastasize, intermediate and high-grade lesions may develop metastases in approximately 16% to 38% of cases [93–95]. Importantly, low-grade myxofibrosarcomas may become higher grade in subsequent recurrences and acquire metastatic potential. The overall 5-year survival rate is 60%–70% [13].

**Figure 4:**
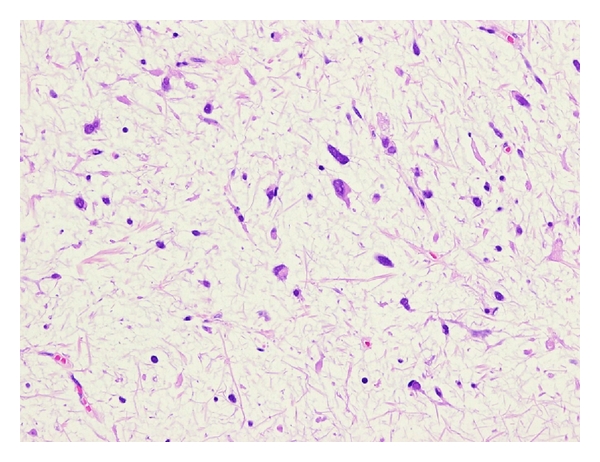
Myxofibrosarcoma with a myxoid stroma containing spindle or stellate-shaped cells with mildly atypical, hyperchromatic nuclei.

 Histologically, myxofibrosarcoma is characterized by multinodular growth of spindle or stellate-shaped cells within variably myxoid stroma containing elongated, curvilinear blood vessels (Figure 4). The tumor cells have slightly eosinophilic cytoplasm and mildly atypical, hyperchromatic nuclei. Vacuolated cells with cytoplasmic acid mucin, mimicking lipoblasts, are also seen [13]. Mitotic figures are rare in low-grade lesions. In contrast, high-grade myxofibrosarcomas are composed of solid sheets and fascicles of atypical spindled and pleomorphic tumor cells with hemorrhagic and necrotic areas. Bizarre, multinucleated giant cells are also occasionally found. Mitotic figures, including abnormal mitoses, are frequent. At least focally, however, areas of a lower grade neoplasm with a prominent myxoid matrix are present [13]. Intermediate-grade myxofibrosarcomas are more cellular than low-grade lesions and often contain minute solid areas showing flank pleomorphism. Immunohistochemically, the tumor cells are diffusely positive for vimentin and occasionally for muscle specific actin and *α*-smooth muscle actin, suggestive of focal myofibroblastic differentiation.

 Data on the cytogenetics and molecular genetics of myxofibrosarcoma are difficult to evaluate, because the diagnostic criteria for this tumor have changed with time. In general, myxofibrosarcomas are associated with highly complex karyotypes lacking specific structural aberrations [96–98]. The only recurrent gain involves chromosome 7, whereas losses primarily affect chromosomes 1, 3, 5, 6, 10, 12, 16, 17, and 19 [7]. The presence of ring chromosomes has been described in some cases of low-grade myxofibrosarcoma (or myxoid MFH) [98–100]. In addition, homogeneously staining regions, double minutes, and marker chromosomes have been found. Recently, Willems et al. [98] proposed the concept of progression of myxofibrosarcoma as a multistep genetic process ruled by genetic instability.

 A conventional CGH study of 22 myxofibrosarcomas showed gains of 19p and 19q, losses of 1q, 2q, 3p, 4q, 10q, 11q, and 13q, and high-level amplifications of the central regions of chromosome 1, 5p, and 20q [101]. Interestingly, gain of 5p and loss of 4q are not observed in low-grade myxofibrosarcomas as opposed to higher grade neoplasms, suggesting that these aberrations are late events in the oncogenesis of myxofibrosarcoma. In addition, array CGH studies showed gains of 7p21-22, 7q31–35, and 12q15–21 and losses of 10p13-14, 10q25-26, and 13q14–34 [38, 102, 103]. These findings suggest that loss of chromosome 13q is the most frequent genomic imbalance in myxofibrosarcoma, leading to inactivation of the RB pathway.

Recently, Lee et al. [103] reported that MET is expressed in approximately two-third of cases and its overexpression is highly related to deep location, higher grades, and more advanced stages. The authors suggested that MET may represent a target of choice to develop novel therapeutic strategies for myxofibrosarcoma.

 A recent gene expression analysis has shown that the *WISP2*, *GPR64*, and *TNXB* genes are upregulated in myxofibrosarcoma compared with other spindle cell and pleomorphic sarcomas [104]. *WISP2* encodes a member of the WNT1 inducible signaling pathway protein subfamily, which belongs to the connective tissue growth factor family. WISP2 is a secreted protein involved in several important human diseases or conditions that are marked by aberrant cell proliferation and migration [105]. GPR64 is a highly conserved, tissue-specific, seven-transmembrane receptor of the human epididymis [106]. *TNXB* encodes a member of the tenascin family of extracellular matrix glycoproteins. TNXB is thought to function in matrix maturation during wound healing, and its deficiency is associated with the connective tissue disorder Ehlers-Danlos syndrome [107]. Nakayama et al. [104] suggested that these genes may serve as novel diagnostic markers for myxofibrosarcoma. Most recently, Barretina et al. [44] demonstrated that *NF1* is mutated or deleted in 10.5% of myxofibrosarcomas.

## 7. Myxoinflammatory Fibroblastic Sarcoma

Myxoinflammatory fibroblastic sarcoma (MIFS), also known as inflammatory myxohyaline tumor of the distal extremities with virocyte or Reed-Sternberg-like cells, is a recently described soft-tissue tumor entity [108, 109]. MIFS occurs predominantly in the subcutaneous tissues of distal extremities and has a peak incidence in the fourth and fifth decades of life with no gender predilection. Patients typically present with a slowly growing, painless, ill-defined mass. The preoperative diagnosis in most cases is benign and may include tenosynovitis, ganglion cyst, and giant cell tumor of tendon sheath [13]. Local recurrence and metastatic rates are 31.3% and 3.1%, respectively [110].

**Figure 5:**
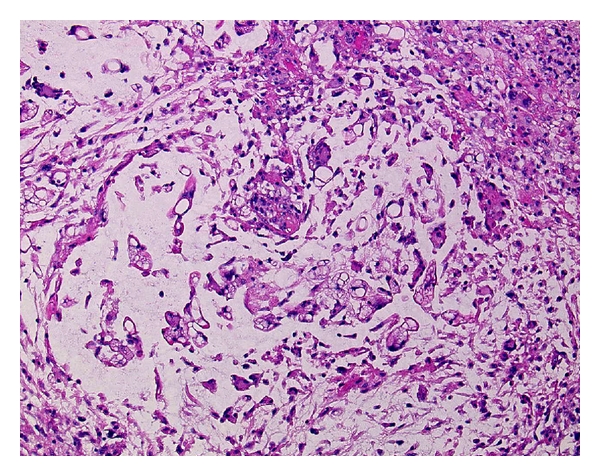
Myxoinflammatory fibroblastic sarcoma with a myxoid background containing spindle-shaped and epithelioid cells, inflammatory cells, and pseudolipoblasts.

 Histologically, MIFS is multinodular, poorly delineated, and characterized by a prominent myxoid matrix containing numerous inflammatory cells, including lymphocytes, plasma cells, neutrophils, and eosinophils [109]. Germinal centers are occasionally encountered. Neoplastic cells include spindle-shaped and epithelioid cells with mild to moderate nuclear atypia, large polygonal and bizarre ganglion-like cells, Reed-Sternberg-like cells with huge inclusion-like nucleoli, and multivacuolated lipoblast-like cells (Figure 5). Hemosiderin deposition may be conspicuous. Mitotic activity is usually low, and necrosis is rarely present. Immunohistochemically, the tumor cells are diffusely positive for vimentin and focally for CD68 and CD34 [16]. Occasional cases may show scattered cells that stain for cytokeratin or *α*-smooth muscle actin. Immunostains for S-100 protein, HMB-45, desmin, EMA, leukocyte common antigen, CD15, and CD30 are typically negative.

 Cytogenetic and molecular cytogenetic studies have identified the frequent presence of a balanced or unbalanced *t*(1; 10)(p22; q24) translocation and ring chromosomes containing amplified material from the 3p11-12 region in MIFS [111–113]. A balanced translocation, *t*(2; 6)(q31; p21.3), has also been described as the sole anomaly in a single case [114]. Most recently, Antonescu et al. [115] demonstrated the presence of *TGFBR3* (1p22) and *MGEA5* (10q24) gene rearrangements by FISH in a subset of MIFS. It is of interest that the *t*(1; 10) translocation and these gene rearrangements have also been identified in hemosiderotic fibrolipomatous tumor (HFLT) [113, 115–117]. These findings suggest that MIFS and HFLT may represent different morphologic variants of the same entity.

Conventional and array CGH studies showed amplification of 3p11-12 [113, 118]. Notably, Hallor et al. [113] demonstrated that 3p11-12 amplification is associated with an increased expression of *VGLL3* and *CHMP2B*. *VGLL3* encodes a protein that is a cofactor of transcription factors of the TEAD family. It has also been shown that *VGLL3* is amplified and overexpressed in myxofibrosarcoma, undifferentiated pleomorphic sarcoma, and dedifferentiated liposarcoma [119]. These findings strongly suggest that *VGLL3* is the main target of 3p11-12 amplification and this genetic event plays an important role in the development and progression of certain subsets of soft-tissue sarcomas.

A recent gene expression analysis has shown that the *FGF8* and *NPM3* genes are upregulated in the *t*(1; 10) -positive tumors compared with tumors without such a translocation [113]. These two genes downstream of *MGEA5* have been mapped to 10q24. FGF8, a member of the fibroblast growth factor family, is a secreted heparin-binding protein, which has transforming potential. FGF8 is widely expressed during embryonic development. Overexpression of *FGF8* has been shown to increase tumor growth and angiogenesis [120]. Hallor et al. [113] suggested that deregulation of *FGF8* may constitute an important event in the development of a subset of MIFS.

## 8. Myxoid Dermatofibrosarcoma Protuberans

Myxoid dermatofibrosarcoma protuberans (DFSP) is a rare but distinctive variant of DFSP with a prominent myxoid matrix. Clinically, myxoid DFSP is similar to typical DFSP [121–123]. DFSP occurs primarily young to middle-aged adults with a male predominance, but this tumor may affect children, including congenital occurrence [124]. It typically presents as a slowly growing, plaque-like or small nodular lesion. The most common location is the trunk, followed by the limbs and head and neck. Local recurrence and metastatic rates are 0%–52% and 0%–1.7%, respectively [125]. The overall prognosis of typical DFSP is excellent if completely excised with negative microscopic margins. Reimann and Fletcher [122] stated that myxoid DFSP appears to have a similarly good prognosis. Recognition of this DFSP variant is important to avoid misdiagnosis of more or less aggressive myxoid soft-tissue tumors.

**Figure 6:**
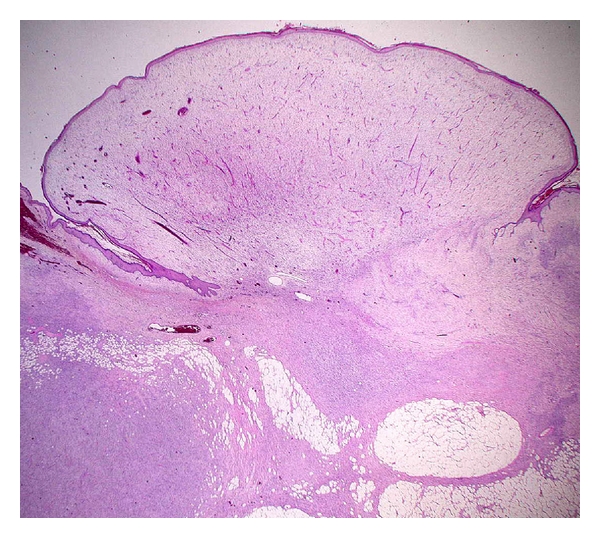
Typical example of a myxoid dermatofibrosarcoma protuberans.

 Histologically, myxoid DFSP is characterized by a sheet-like to vaguely lobular proliferation of bland spindle cells in an abundant myxoid stroma (Figure 6). The tumor cells have slightly eosinophilic cytoplasm and stellate to oval nuclei with indistinct nucleoli. Branching, thin-walled blood vessels are frequently present. All cases display at least focally a strikingly infiltrative growth pattern, with trapping of subcutaneous adipose tissue in the characteristic honeycomb manner also observed in typical DFSP [122]. Mitotic activity is usually low. Immunohistochemically, the tumor cells are diffusely positive for vimentin and CD34. Immunostains for S-100 protein, desmin, muscle specific actin, *α*-smooth muscle actin, cytokeratin, and EMA are typically negative. Apolipoprotein D (APOD) has been found to be highly expressed in DFSP and its histological variants [126].

 DFSP is characterized by an unbalanced translocation *t*(17; 22)(q22; q13), which fuses the *COL1A1* gene on 17q21-22 with the *PDGFB* gene on 22q13 [127–130]. The same molecular event is also seen in supernumerary ring chromosomes derived from the *t*(17; 22) [129, 130]. Identical genetic changes have also been shown in the histological variants, including myxoid DFSP [123], pigmented DFSP (Bednar tumor) [131], Granular cell DFSP [132], juvenile variant of DFSP (giant cell fibroblastoma) [128], and fibrosarcomatous variant of DFSP [133, 134]. Other rare translocations, including *t*(*X*; 7), *t*(2; 7), *t*(9; 22), and *t*(5; 8), have also been described [135–138]. Moreover, several secondary nonrandom alterations have been identified, including trisomy 5 and trisomy 8 [130]. The clinical and biological implications of these chromosomal alterations are virtually unknown.

 Conventional and array CGH studies showed gain or high-level amplification of 17q and 22q in most cases [139–141]. DFSP is occasionally misdiagnosed as benign lesions such as dermatofibroma, leading to improper primary management. We suggested that CGH may be a useful diagnostic tool for distinguishing DFSP from dermatofibroma [140]. The presence of gain in 8q was also observed [140–142]. Interestingly, FISH and CGH studies have indicated an association between an increased number of *COL1A1-PDGFB* genomic copies and fibrosarcomatuos transformation in a subset of DFSP [139, 143, 144]. Most recently, Salgado et al. [145] reported that the majority of DFSP harbor the *COL1A1-PDGFB* fusion and FISH should be recommended as a routine diagnostic tool.

 The breakpoint of *PDGFB* is remarkably constant (exon 2). In contrast, the *COL1A1* breakpoint may occur in any of the exons in the *α*-helical coding region (exons 6–49). The most frequently rearranged *COL1A1* exons are exon 25, 32, and 47 [146]. *PDGFB* encodes the *β* chain of platelet-derived growth factor. PDGFB is a potent mitogen for a variety of cells [147]. *COL1A1* encodes the pro-*α*1 chains of type I collagen whose triple helix comprises two *α*1 chains and one *α*2 chain. Type I collagen is a major structural protein found in the extracellular matrix of connective tissue such as skin, bone, and tendon. The COL1A1-PDGFB fusion protein is posttranslationally processed to a functional PDGFB, and results in PDGFB-mediated autocrine and/or paracrine activation of PDGFRB [128, 148]. Inhibitors of PDGFRB, such as imatinib mesylate, have been reported to show clinical activity for metastatic or locally advanced DFSP [149–151]. These results support the concept that DFSP cells are dependent on aberrant activation of PDGFRB for cellular proliferation and survival. No correlation between the molecular subtype of *COL1A1-PDGFB* fusion gene and the clinicopathological features has been established [146, 152].

 Gene expression profiling studies of DFSP have revealed overexpression of the *PDGFB*, *PDGFRB*, *APOD*, *SPRY2*, *NRP1*, *EGR2*, and *MEOX1 *genes [10, 153]. *SPRY2* encodes a protein belonging to the sprouty family and is involved in the regulation of the EGF, FGF, and Ras/MAPK signaling pathways. NRP1 is a membrane-bound coreceptor to a tyrosine kinase receptor for both vascular endothelial growth factor and semaphorin family members and plays a role in angiogenesis, cell survival, migration, and invasion. EGR2 is a transcription factor with three tandem C2H2-type zinc fingers and plays a role in the PTEN-induced apoptotic pathway [154]. *MEOX1* has been mapped to 17q21 and encodes a member of a subfamily of nonclustered, diverged, antennapedia—like homeobox—containing genes. The homeobox genes are involved in early embryonic development and the determination of cell fate. Linn et al. [153] proposed the possibility that DFSP are derived from early embryonic mesenchymal cells.

## 9. Conclusions

It is important to be familiar with the clinicopathological and molecular genetic features of myxoid soft-tissue sarcomas for their accurate diagnosis and appropriate treatment. In our experience, FISH is a valuable ancillary diagnostic tool for these sarcomas, especially on limited tissue samples. Novel diagnostic and/or prognostic molecular markers as well as promising therapeutic targets have gradually been recognized. In the future, treatment decisions and prognosis assessment for myxoid soft-tissue sarcomas will increasingly be based on a combination of histological criteria and molecular identification of genetic alterations indicative of biological properties.

## Figures and Tables

**Table 1:** Chromosomal alterations and related molecular events in myxoid soft-tissue sarcomas.

Tumor type	Chromosomal alteration	Molecular event	Prevalence
Myxoid/round cell liposarcoma	*t*(12; 16)(q13; p11)	*FUS-DDIT3*	>90%
	*t*(12; 22)(q13; q12)	*EWSR1-DDIT3*	<5%
Low-grade fibromyxoid sarcoma	*t*(7; 16)(q32–34; p11)	*FUS-CREB3L2*	>95%
	*t*(11; 16)(p11; p11)	*FUS-CREB3L1*	<5%
Extraskeletal myxoid chondrosarcoma	*t*(9; 22)(q22; q12)	*EWSR1-NR4A3*	75%
	*t*(9; 17)(q22; q11)	*TAF15-NR4A3*	15%
	*t*(9; 15)(q22; q21)	*TCF12-NR4A3*	<1%
	*t*(3; 9)(q12; q22)	*TFG-NR4A3*	<1%
Myxofibrosarcoma	Complex karyotype	Not known	Not applicable
Myxoinflammatory fibroblastic sarcoma	*t*(1; 10)(p22; q24)	Deregulation of *FGF8* and *NPM3 *	Not applicable
		Rearrangement of *TGFBR3* and *MGEA5 *	Not applicable
Myxoid dermatofibrosarcoma protuberans	*t*(17; 22)(q22; q13)*	*COL1A1-PDGFB*	>90%

*Rearrangement also frequently seen as a ring chromosome.
